# Watch and Learn: Vicarious Threat Learning across Human Development

**DOI:** 10.3390/brainsci11101345

**Published:** 2021-10-13

**Authors:** Yael Skversky-Blocq, Jan Haaker, Tomer Shechner

**Affiliations:** 1 School of Psychological Sciences and the Integrated Brain and Behavior Research Center, University of Haifa, Haifa 3498838, Israel; yskversk@gmail.com; 2 Department of Systems Neuroscience, University Medical Center Hamburg-Eppendorf, 20251 Hamburg, Germany; j.haaker@uke.de

**Keywords:** vicarious threat learning, observational learning, child development, adolescents, fear

## Abstract

Vicarious threat learning is an important pathway in learning about safety and danger in the environment and is therefore critical for survival. It involves learning by observing another person’s (the demonstrator) fearful responses to threat and begins as early as infancy. The review discusses the literature on vicarious threat learning and infers how this learning pathway may evolve over human development. We begin by discussing the methods currently being used to study observational threat learning in the laboratory. Next, we focus on the social factors influencing vicarious threat learning; this is followed by a review of vicarious threat learning among children and adolescents. Finally, we examine the neural mechanisms underpinning vicarious threat learning across human development. To conclude, we encourage future research directions that will help elucidate how vicarious threat learning emerges and how it relates to the development of normative fear and pathological anxiety.

## 1. Introduction

Early in life, children learn to differentiate threat and safety in their environment. Direct and vicarious learning are two pathways by which people acquire new threat associations [1]. *Direct threat learning* is based on Pavlovian classical fear-conditioning [2]. In classical conditioning, a person experiences an aversive stimulus (i.e., dog bite) that is then associated with a neutral stimulus (i.e., dog), such that a threat response is elicited by the neutral stimulus alone (i.e., fear of the dog). *Vicarious threat learning* is based on social learning theory [3], which emphasizes how people learn from *social* experiences in the environment. Two types of vicarious learning are commonly discussed: observational and verbally instructed. In observational threat learning, a person watches another person (the demonstrator) experiencing an aversive event or expressing fear and avoidance of a threat. By watching this person, the observer learns that a specific stimulus is dangerous without directly experiencing the threat. In verbally instructed threat conditioning, a person receives verbal information about a threat (e.g., a type of dangerous animal), and learning occurs without observing others’ responses to the threat.

Threat conditioning literature focuses mainly on direct learning using classical threat conditioning, when, in fact, most early threat learning experiences are not based on direct but vicarious experiences [1,4,5,6]. Toddlers do not need to suffer a bad burn to know fire is dangerous. They will learn to be careful after receiving multiple warnings from their caretaker not to approach the stovetop. Further, children may be nervous around spiders after observing a parent’s explicit fearful response. While vicarious threat learning is an essential pathway to learn about threat and safety during development, very little laboratory-based research has focused on this type of learning, particularly among youth.

This paper reviews findings in observational threat learning research conducted in children and adolescents. This is the first review to focus specifically on developmental vicarious threat learning [4,7] and to emphasize recent literature in the field [8,9]. Given the limited findings, we use data from observational threat learning studies conducted with adults, as well as relevant findings from more general research on social and emotional development. Our goal is to integrate various aspects of development and to infer how they may influence vicarious threat learning across the different stages of maturation. Importantly, this review focuses solely on human vicarious threat learning, complementing a recent review of work on non-human animals [7].

The review unfolds as follows. We begin by describing the methods and measures used to study observational threat learning in the laboratory. Next, we discuss the social factors influencing vicarious threat learning and review the literature on vicarious threat learning among youth. We then turn to the impact of brain development on social threat learning. Finally, we focus on vicarious safety learning to highlight the counter-effects of positive modeling on learned-threat associations. To conclude, we consider methodological and critical ethical challenges for conducting vicarious threat learning studies with youth and encourage future research directions that will help elucidate how vicarious threat learning emerges and how it relates to the development of normative fear and pathological anxiety.

## 2. Methods and Measurements of Vicarious Threat Learning in Developmental Studies

Vicarious threat learning has been studied in the laboratory using a variety of methods and measures. One common method is to use pre-recorded standardized videos of a learning model undergoing a direct threat-learning task [9,10,11,12]. A recently published protocol allows better standardization and reproducibility of results using pre-recorded videos of a learning model (a stranger), a method often applied in adult populations [10]. One study in children included prerecorded videos of an adult learning model, either a parent or a stranger [11], while another study used an adolescent learning model (14 years old) to investigate children (8–13 years), adolescents (13–17 years), and adults (18–38 years) [9]. These pre-recorded videos have unanimously induced fear via observation in adults, adolescents, and children.

A second method to study vicarious threat learning employs images of adults or peers displaying scared or neutral facial expressions associated with images of novel neutral stimuli [13,14,15]. This method has been successfully applied with school-aged children (7–10 years), resulting in children reporting greater fear beliefs for scared-paired novel stimuli than neutral-paired novel stimuli. However, to induce sustained fear in young children, prior negative verbal information about the stimuli is recommended to augment the observational learning [4,13,14,16,17,18,19,20].

A third method uses real-time procedures, thereby enhancing the ecological validity of laboratory vicarious threat learning [21,22,23,24]. This method includes parent or stranger adult learning models undergoing a direct threat learning task. Participants observe the learning model in real time, rather than in standardized pre-recorded videos. Several of these studies have been conducted with infants (12 months) and toddlers (15–20 months), highlighting that even at a young age, children can learn fear and avoidance by watching their mother’s fearful facial expressions [21,22]. Real-time vicarious learning paradigms with adult and child observers have yielded similar results. One study have shown that observers learned differential fear by watching a live demonstrator undergoing direct threat learning. In addition, greater observer–demonstrator physiological synchrony during learning predicted higher physiological arousal when the observer was later directly exposed to the stimulus [23]. Another study with friend dyads during a real-time vicarious procedure demonstrated that differential learning occurred as a function of CS-US contingency awareness. Observers who understood CS-US contingency after watching their friend, showed greater differential physiological arousal during the later direct exposure phase than those who did not understand CS-US contingency [24]. Importantly, many of the aforementioned procedures have been used in studies conducted with adults, and some have been specifically designed for youth. Suggestions on methodology and paradigms for vicarious threat learning are discussed at the end of the review.

Most vicarious threat learning studies, regardless of the procedure, use at least two phases: an observational threat learning phase (demonstrator is present) followed by a direct exposure test phase (demonstrator is absent). Multiple physiological and self-report measures are applied in both phases. Physiological measures include skin conductance response (SCR), fear-potentiated startle (EMG), functional magnetic resonance imaging (fMRI), and eye-tracking. Self-report measures include fear ratings, US expectancy, risk assessment, and CS-US contingency. Studies using these different measures are reviewed in the following sections.

## 3. Factors Contributing to Vicarious Threat Learning

As vicarious threat learning is inherently social, features of the learner (observer), the model (demonstrator), and their interaction all influence learning. Most of the studies examining these factors have been conducted with adults. The next section reviews this literature and infers how these features may differ across development.

### 3.1. Observer Factors

#### 3.1.1. Empathy

While it is important to understand the emotional response of the demonstrator when responding to a threat (sympathy), it is unclear if sharing these emotional responses with the demonstrator (empathy) [25,26] is essential for vicarious threat learning. Extensive research on the influence of empathy on observational threat learning has examined its role but has yielded mixed results. Some studies demonstrate vicarious threat learning is influenced by observer empathy [27,28], whereas others show empathy and vicarious threat learning are not necessarily linked [29]. One study found directing participants to enhance their empathy for the demonstrator resulted in greater physiological arousal during a later direct exposure test. In addition, this effect was augmented by higher trait empathy among observers [28]. Another study reported similar findings, showing that higher trait empathy was associated with prolonged gaze toward the threat cue during observational acquisition [27]. A recent study replicated the procedure reported by Olsson et al. [28], directing participants to enhance their empathy for the demonstrator during a vicarious threat conditioning task. Surprisingly, this study could not replicate the previous findings, showing instead that cognitive and affective empathy did not amplify physiological arousal during vicarious threat learning [29]. This discrepancy may be explained by methodological differences, such as the use of different empathy scales. However, it also highlights that differences in the ability to share the emotional response of the demonstrator (empathy) might not modulate observational threat learning in a salient way. Rather, the mere information about the intensity of the demonstrator’s emotional response (sympathy) modulates the strength of observational threat learning in adults [30], as well as in children [31].

If, in fact, sympathy is sufficient to induce observational threat learning, then even young children who do not necessarily share the demonstrator’s emotion but still understand how the demonstrator feels are able to learn fear vicariously; and, indeed, at 15 months of age, toddlers can already understand basic emotions demonstrated by their caregiver’s facial expressions, and thus can learn, vicariously, to associate different emotional expressions with safe and dangerous stimuli in the environment [21].

The development of empathy, as well as social cognition in general, and theory of mind may further inform differences in vicarious threat learning among youth. Pre-school children begin to take another person’s perspective (theory of mind) and have greater cognitive understanding of other people’s emotional states [32]. By school age, they can take another person’s perspective and verbally articulate how that person may be feeling [33]. These social cognition skills develop even more during early and late adolescence [34]. As their emotional range and social understanding grow, children’s ability to recognize subtle emotional expressions also matures, making them better equipped to learn more complicated vicarious threat responses.

#### 3.1.2. Sex Differences

Sex differences have seldom been reported in human vicarious threat learning [7,35,36]. One study found that after watching either a male or female demonstrator having panic attacks associated with the CS+ but not the CS−, female observers reported more distress and greater dislike toward the CS+ relative to the CS− than male observers. In addition, female observers believed the female demonstrator displayed more panic attack symptoms than the male observers did. Although significant gender differences were revealed in the self-reports, this study did not find sex differences in physiological arousal [35]. Another study with adults examined the effect of sex hormones on vicarious threat learning [36]. Estrogen and testosterone were administered to male and female participants, respectively, who were then compared to male and female controls in their ability to recognize social cognition and to learn threat via observation. Testosterone-treated women were less accurate at recognizing social intentions and emotions than control-treated women, while estrogen-treated males showed higher SCR toward the social US during observational threat learning than control-treated males. In other words, testosterone can impact women’s social-cognitive processing, and estrogen can increase men’s autonomic reactivity to seeing another person’s distress.

Recently, Reynolds et al. [37] found an interesting developmental (7–11 years) sex difference during vicarious threat learning. Boys perceived fearful faces as significantly more fearful than girls, but girls perceived neutral faces as significantly more fearful than boys. This difference, however, did not translate to sex differences in differential vicariously learned threat. Another study found a strong sex difference in very young children. Female toddlers showed more avoidance behavior than male toddlers after watching their mothers’ fear/disgust expressions [21]. While the specific reason for this sex difference was not directly examined, the researchers hypothesized that the congruence of the toddler’s and mother’s sex may have played a role in increasing sensitivity to the model.

#### 3.1.3. Physiology and Biology

Specific physiological and biological factors impact observational threat learning. A recent study found observer sleepiness increased vicariously learned fear cognitions, avoidance, and attentional bias toward threat in a youth sample [37]. As mentioned previously, changes in sex hormones can affect social evaluation and vicariously learned threat [36]. Another study on adults showed that blocking the release of endogenous opioids increased the observer’s response to watching the demonstrator’s distress and produced a long-term threat response following observational threat learning [38,39].

In brief, vicarious threat learning is a complex pathway to learn about danger and safety; more research is required on humans, especially youth, to understand the biological and physiological factors at play.

### 3.2. Demonstrator Factors

#### 3.2.1. Anxiety

Demonstrator factors have been shown to moderate vicarious threat learning. For example, a demonstrator’s explicitly expressed pain or anxiety is likely to influence vicarious threat learning. One study showed that participants learned to discriminate between safe and dangerous stimuli similarly from either an anxious or a non-anxious demonstrator. However, those who learned from the anxious demonstrator showed slower extinction, displaying a prolonged discrimination between the safety and danger cues, than those who learned from a non-anxious demonstrator [40]. A recent study found parental anxiety level was positively associated with children’s (6–17 years) ability to differentiate between the safety and danger cues [8]. This finding is in line with other developmental research showing that a mother’s socially anxious behavior generates greater social wariness in infants [22,41].

#### 3.2.2. Trustworthiness

Higher demonstrator credibility, authority, and skill may have a profound effect on vicarious threat learning as well [42]. Although no studies have directly manipulated these demonstrator factors in vicarious threat learning paradigms, social learning is known to be strongly influenced by the trustworthiness and prestige of the informant [43,44]. More studies should manipulate and explore these demonstrator factors to uncover how demonstrator trustworthiness and credibility may modulate vicarious threat learning.

### 3.3. Interaction of Observer–Demonstrator Factors

Many observer-demonstrator factors have been posited as potential moderators in vicarious threat learning, and more related research has been conducted with youth. These factors can be divided into two categories: (1) similarity (physical and other) between the observer and demonstrator and (2) relatedness, or the relationship between them.

#### 3.3.1. Similarity (Physical and Other)

Age: Age congruency between the observer and demonstrator has not been shown to impact vicarious threat learning in a meaningful way [9,45]. For example, one study found children (6–10 years) learned fear similarly from peer and adult faces expressing fear [45]. In another study, children (8–12 years), adolescents (13–17 years), and adults (18–34 years) showed a similar differential fear response (SCR) while observing a 14-year-old adolescent learning model undergoing direct threat learning [9].

Racial group: Several studies in adults have shown that learning about threat and safety from a racial in-group demonstrator is stronger than learning from a racial out-group demonstrator [46,47]. No developmental studies to date have been conducted with youth specifically examining racial group effects during vicarious threat learning. Nevertheless, abundant research has demonstrated the effects of racial in-group versus out-group on various psychological indices (face processing, attention, memory, etc.). For example, as early as infancy, there is some evidence of racial bias toward in-group faces when learning about cues in the environment [48,49,50,51,52,53,54]. However, the impact of racial similarity between the observer and demonstrator on vicarious threat learning is likely to be different across development.

#### 3.3.2. Relatedness

Familiarity: The relationship between the observer and the demonstrator has been suggested as a significant factor in vicariously learned fear. While no studies on adults have specifically compared stranger-dyads with friend-dyads, one real-time vicarious threat learning study found friends were able to learn differential fear by watching another friend undergoing a direct-threat-learning task [24]. Extant research has also shown that observing a close friend in pain or embarrassment generates more empathy and stronger neural activity in affect-related and mentalizing brain regions [55,56].

Parents: Parent–child paradigms are becoming more prevalent in developmental vicarious threat learning studies [11,57,58]. This dyad is especially important for understanding developmental vicarious threat learning, as the parent or primary caretaker is the first social agent and thus the first model for a child to learn about the world. Two studies found children (6–12 years) learned differential fear similarly from either a parent or a stranger demonstrator [11,57]. In contrast, another study found stronger differential observational threat learning from ‘own parent’ demonstrators than from ‘unfamiliar parent’ demonstrators in youth (6–17 years) observers [8]. Another study found that, if the father–child relationship was characterized by insecure attachment, children seemed to be more anxious and more vulnerable to threat learning modeled by their fathers [58].

Alliance: Shared or opposing opinions among observers and demonstrators also moderate vicarious threat learning. One study found individuals from a racial in-group who held similar opinions (e.g., rooting for the same sports team) exhibited greater differential fear responses than those from a racial in-group who did *not* hold similar opinions (e.g., rooting for a rival sports team) [59]. This result suggests physical similarity is influential in social fear learning; however, whether it increases or decreases that fear may hinge on shared alliances and feelings of ‘sameness’ between the observer and the demonstrator.

## 4. Vicarious Threat Learning across Development

Observational threat learning occurs from the moment we are born and continues throughout our lifetime [4,7,12]. Yet, only in the last 20 years have researchers begun to systematically study children and adolescents in prospective and real-time procedures to understand how vicariously learned fear interacts with development.

Modeling is a powerful way for youth to learn about what is safe versus dangerous in the environment [4,9,11,15,21]. In several studies, school-aged children (7–10 years) who completed a vicarious-threat-learning task reported greater fear beliefs, showed more avoidance of fearful stimuli, and had a higher heart rate than children who did not complete the learning task [13,14,15,57]. Recently, school-aged children (8–12 years) were shown to exhibit differential (CS+ vs. CS-) fear responses after watching a video of a learning model undergoing a direct-threat-learning task [9,11]. Children exhibited stronger physiological responses to the threat cue than the safety cue after observing a parent, an adult stranger, or a peer stranger undergoing direct threat conditioning. Interestingly, one study found that although children responded differentially in physiological outcome measures (e.g., SCR), they were unable to articulate this differential fear in self-reports [9]. This finding emphasizes a developmental nuance in vicarious threat learning, specifically for school-aged and possibly even younger children.

More research is needed on vicarious threat learning among adolescents, given the salience of social factors in this age group [60,61]. While some studies have examined social learning and the intergenerational transmission of anxiety in adolescents [62,63], only one study to date has investigated observational threat learning specifically in this age group [9]. This study found adolescents (13–17 years) learned differential fear by watching an adolescent demonstrator, as indicated by their differential SCR to the threat and safety cues. Yet, adolescents tended to generalize their fear more than adults when they reported differential fear.

Although progress has been made in understanding vicarious threat learning across development, certain key methodological limitations remain. Many paradigms use static images rather than live models and augment observation with explicit instruction. In addition, only in the last few years have researchers begun measuring heart rate, skin conductance, and other autonomic responses in youth, in addition to self-reported fear beliefs. These studies have uncovered important knowledge about what happens physiologically during developmental vicarious fear learning, but more research is needed to expand this expertise, especially on the neural level.

## 5. Neural Underpinnings of Vicarious Threat Learning

In the last decade, studies have explored the neural underpinnings of direct and vicarious threat learning in adults [7,64]. These studies have revealed overlapping neural activation in observational and direct threat learning but diverging circuit connectivity.

Several brain regions have been linked to both direct and vicarious threat learning in adults. The amygdala, considered a central hub in the processing of threats [65] and social information [66,67], is active during both direct and observational threat learning [38,68,69]. Increased activity in the anterior insula (AI), together with the dorsal anterior cingulate cortex (dACC), has also been associated with direct [70] and vicarious threat learning [38]. The finding of the involvement of the AI and dACC in vicarious threat learning is in line with robust evidence indicating the recruitment of these regions when observing another person’s response to pain [71,72,73]. In addition to these forebrain regions, the periaqueductal grey (PAG), which is critical for autonomic, behavioral, and anticipatory responses, has been found active in direct and observational threat learning [38,74].

At the same time, disparate neural connectivity has been noted in studies comparing direct and observational threat learning [68]. Specifically, one study found the temporo-parietal junction (TPJ), implicated in social cognition and theory of mind [75], had a stronger association with the anterior insula during observational learning than direct learning. This connection between the TPJ and the AI suggests encoding a social US entails a linkage between processing another person’s emotional state (TPJ) and processing an aversive stimulus (AI). In the same study, the AI was positively correlated with greater empathy for the demonstrator, more discomfort when observing the demonstrator’s pain, and greater perceived intensity of the social US [68]. Finally, greater activity in the superior temporal sulcus (STS), known for its role in audiovisual integration, and also in the dorsomedial prefrontal cortex (dmPFC), known for its multimodal role in social and emotional information processing, has been linked to social threat learning [64,68,76,77,78].

Interestingly, the overlap in neural activation between observational and direct threat learning does not imply similar processing. In fact, the neural connectivity during both direct and observational threat learning has revealed different regions providing input into an overlapping network (dACC, AI, amygdala): the amygdala is the likely input region during direct threat learning, whereas the AI is more likely the input region during observational threat learning [68]. Of note, many non-human animal studies have confirmed the involvement of these particular neural regions in social threat learning [7,64,79,80,81] and discovered similar distinctions in the circuit encoding of observed aversive outcomes in cortical and amygdala projections [79,82,83].

Finally, somatosensory cortices are activated when people observe the behavioral responses and feelings of others and are commonly associated with vicarious learning [84]. The ‘mirroring’ of activation in primary somatosensory cortices are associated with both first-hand and observational experiences. This is in line with meta-analyses demonstrating overlapping activity in primary somatosensory areas during both first-hand nociception as well as encoding information about pain in others [71,72,73].

Notably, activation in the somatosensory system has been found to interact with processes related to empathy and pro-social behavior during vicarious pain [85,86,87,88]. Moreover, the lower part of the somatosensory system in the spinal cord was shown to be involved during the observation of aversive outcomes. Responses in the dorsal horn, which were positive to direct pain, were in fact negative when the painful stimulus was merely observed [89]. These findings suggest somatosensory representations play a role in vicarious emotional responses, pointing to a unique neural mechanism underlying vicarious fear and threat learning. To the best of our knowledge, only one functional neuroimaging study has examined vicarious threat learning in youth [8]. In the next section, we highlight these findings while also relying on the relevant, albeit more general, research on social brain development in an attempt to infer neural similarities and differences between youth and adults in vicarious threat learning.

### 5.1. Vicarious Threat Learning fMRI Study in Youth

In a recent study, 33 youth participants (6–17 years) watched a video of their own parent demonstrator and a video of an unfamiliar parent demonstrator undergoing differential direct threat learning. Next, participants underwent a direct exposure test. During both phases, fMRI data were collected [8]. Observing own parent demonstrators compared to observing stranger parent demonstrators resulted in less differential amygdala activation (CS+ vs. CS-) during observational acquisition but enhanced differential activation in the test stage. In addition, greater differential learning (CS+ vs. CS-) was evident in self-reports when observing own parent than stranger parent demonstrators. In the next step, fMRI data were collected for a subset of the parents while they were undergoing the direct threat learning task. The results showed parents’ mPFC activation was negatively associated with their child’s amygdala activation during observational acquisition, and their amygdala activation was negatively associated with their child’s mPFC activation during observational acquisition. This finding suggests a moderating effect of parental brain activity on the recruitment of threat-related brain regions in the child during observational threat learning. More research is required to understand whether this is parent-specific or other familiar demonstrators produce similar results in youth observers.

### 5.2. Social Brain Development

In this section, we examine regions active in the adult brain during vicarious threat learning and see how they map to socio-emotional brain development in youth. Social and emotional brain development is likely to influence vicarious threat learning [90,91,92,93]. Multiple pediatric studies have noted amygdala recruitment in the recognition of fearful faces, mirroring findings for adults [94,95,96]. As early as infancy, children are able to recognize and mimic faces and biological motion, both of which rely on cortical structures like the STS [97,98] and the broader somatosensory system [99,100]. As described, the STS has been similarly implicated in adults in audiovisual integration and mentalizing processes during observational threat learning [64]. In the first 3–5 years of life, children begin to understand the intentions of others and can even predict another person’s behavior [101,102]. These mentalizing abilities at an early age involve the medial prefrontal cortex (mPFC), the STS, and other temporal areas (e.g., TPJ) found to be active in adults undergoing social threat learning [103].

A study of 16 youth participants (8–15 years) viewing fearful faces showed a positive correlation between age and heightened prefrontal cortex activity (e.g., different clusters in the middle frontal gyrus and superior frontal gyrus), a region associated with social and emotional control [104]. This finding is in line with an fMRI study comparing pre-adolescents (8–11 years) and adults undergoing a direct threat learning task. Pre-adolescents tended to recruit ‘early-maturing’ subcortical brain regions, such as the amygdala and hippocampus, whereas adults tended to recruit the ‘late-maturing’ dorsolateral prefrontal cortex when discriminating between threat and safety [105]. These two studies highlight that the PFC is particularly plastic during adolescence and therefore may play a different role in generating responses to socioemotional cues among adolescents than among adults [106]. Similarly, in a study evaluating adolescents and adults in their attention to fearful versus neutral faces, adolescents, as compared with adults, showed greater activity in the ACC, the orbital frontal cortex (OFC), and the right amygdala in response to fearful compared to neutral faces [107]. This finding, like the previous finding, emphasizes the heightened brain activity in structures like the amygdala, hippocampus, and ACC in response to social-emotional content, specifically during adolescence [108]. Taken together, the findings suggest that, because socio-emotional cognitions and skills develop parallel to brain maturation, vicarious threat learning likely recruits additional brain regions (presumably prefrontal regions like the ACC) and moderates activity differently throughout development.

## 6. Vicarious Safety Learning

The bulk of this review discusses vicarious threat learning, but the other side of the coin, vicariously safety learning, has important clinical and developmental implications as well. Just as youth learn about threat by observing another person’s fearful response to threat, they can also learn about safety by observing a safe and positive response to a dangerous stimulus. Considerable research with adults and youth has shown that preemptive social safety learning can inhibit threat learning. Moreover, vicarious fear extinction can diminish previously learned threat associations and reduce behavioral avoidance, with long-lasting effects [31,45,57,109,110,111,112,113,114,115,116,117,118,119].

Findings in vicarious safety learning have promising implications for new interventions for anxiety related disorders. One study found observational extinction was more effective in reducing conditioned threat responses than direct extinction, after a direct conditioning task [113]. This finding was extended by Pan et al. [116], who showed the combination of direct extinction and observational extinction (shared extinction) was more effective for reducing fear and avoidance than either direct or observational extinction alone. Interestingly, a study in children (7–9 years) found vicarious threat extinction and verbally-transmitted safety information were equally effective in reducing vicariously-learned fear [119]. These studies are just a few of the many that show vicarious safety learning is a viable and compelling pathway to learn about safety and extinguish fears. A new clinical intervention, ‘observational exposure therapy,’ may be an exciting avenue to pursue, as it would allow more feasible exposures and potentially even better treatment outcomes than current methods of exposure to reduce learned fear and anxiety.

## 7. Vicarious Transmission of Psychopathology

Another aspect related to vicarious threat learning is the vicarious transmission of psychopathology, specifically anxiety. This topic has been well-studied [62,120,121,122,123] and previously reviewed [124]. An important twin study found environmental factors, such as modeling, have more weight than genetic factors in the transmission of anxiety disorders [62]. This finding suggests anxiety may be passed from parent to child through learning experiences rather than biological inheritance. Some studies have demonstrated anxious modeling could be a risk factor for the development of anxiety and fearful behaviors [6,125,126]. For example, parental modeling of social anxiety was associated with infants and young children displaying greater fear and avoidance of strangers [22,41,122,123]. This finding was supported by retrospective studies showing that parental isolation and avoidant behavior preceded their children’s development of social fears and anxious avoidance [127,128]. Interestingly, children’s anxiety was also shown to exacerbate anxious parenting behaviors, pointing to the bi-directional relationship between parental and child anxiety [129,130,131]. Yet, no study to date has examined the long-term effects of anxious modeling on anxiety disorders in youth. Linking early vicarious threat learning processes with the development of anxiety is a valuable area of research due to its clinical implications. If anxious modeling is a risk factor for psychopathology, then early interventions aimed at decreasing anxious modeling or counteracting its negative effects with positive modeling may help prevent the onset of anxiety and its related disorders.

## 8. Final Thoughts and Future Directions

This review has focused on human vicarious threat learning across development, drawing on the relatively limited research conducted with youth and relying heavily on vicarious learning research with adults. The aim was to review the existing literature and to encourage developmental research in the field of vicarious threat learning. As evident in the different sections of the review, social and cognitive development play a central role in how children learn fear vicariously as they grow from infancy to adolescence. Moreover, neural disparities between youth and adults seem to influence vicarious threat learning differently across the lifespan. As socioemotional, cognitive, and neural systems are forming and constantly evolving during development, vicarious threat learning may be uniquely activated and invariably changing during this sensitive period of maturation.

We have not review other important areas of research related to vicarious threat learning as they have already been reviewed. Abundant research has focused on non-human animal models, and a thorough discussion of vicarious threat learning in non-human animals appears in Debiec and Olsson [7]. To this previous work, we add a non-exhaustive review of vicarious threat learning across human development.

In what follows, we discuss issues related to vicarious threat learning across development, including critical ethical challenges of conducting research with youth. We also make suggestions for future developmental research in both laboratory and clinical contexts.

Methodological and ethical challenges: Studying threat learning in youth has inherent methodological and ethical challenges. One critical methodological question is whether to use only observational cues or to augment them with preemptive verbal information, such as informing participants about the negative nature of the CS prior to observational learning [14,16]. Although children may have difficulty articulating vicarious threat learning using only observational cues [9], enhancing learning with explicit verbal instruction might confound the purely observational learning process. Therefore, including or excluding verbal instruction in developmental research on vicarious fear learning depends on the specific question being asked. For example, if the research question concerns the learning process in and of itself, it may be better to use only observational cues and exclude any explicit verbal instruction. In contrast, if the research question concerns downstream effects such as extinction, generalization, or avoidance, it may be helpful to augment the observational learning with verbal information to ensure vicarious threat learning occurs. It should be noted that isolated observational threat learning may not occur ecologically. However, in a laboratory setting, it may be helpful to parse out the two vicarious learning pathways to explore specific developmental nuances in observational and verbally instructed threat learning separately.

Another methodological challenge is related to participants’ understanding of the experimental setting. Adult and even adolescent participants most likely have some concept of what a laboratory ‘experiment’ involves. However, younger children may not have this conceptual understanding and therefore may need more explicit guidance during their first foray into a laboratory setting. This challenge is particularly relevant to vicarious threat learning, as participants must understand that the equipment attached to them is similar to the equipment they observed in the learning model video (for example, the US delivery device). In addition, during vicarious threat learning studies, participants must understand that the same stimuli presented to the model in the observation stage are later presented directly to them in the direct exposure test. These two examples require higher cognitive processes in general, especially in new settings, such as the laboratory. Inherent differences obviously exist between threat learning in the real world and threat learning in the laboratory setting. These differences could have a more profound effect on younger age groups, making in-lab developmental comparisons more difficult.

From an ethical standpoint, it can be problematic to use deception with youth during experimental studies [132]. In vicarious threat learning, participants are never directly exposed to the US. They watch another person experiencing an electrical stimulation or a loud aversive sound and are then led to believe they may receive a similar shock or sound application, though participants never receive the US and are, thus, deceived. This deception should be considered carefully, especially in youth, and it goes without saying that studies including deception should always follow all the ethical guidelines, including debriefing participants at the end of the experiment. Parental consent is of utmost importance in all studies with youth, and parents should be notified about any deception in vicarious threat learning before giving their consent.

Paradigm considerations for vicarious threat learning in youth: Research in direct threat learning was first conducted on adults and later adapted for a developing population [133]. There are now numerous studies and paradigms with youth that have contributed to our understanding of how fear develops and what interventions are important for counteracting the negative effects of maladaptive threat associations. Still, there is much heterogeneity in the paradigms used in threat conditioning research among youth and adults, and the field is pushing for standardized rather than novel tasks [134,135]. As in work on direct threat learning, researchers have generated developmentally appropriate vicarious threat learning paradigms [4]. However, while encouraging more research on vicarious threat learning in youth, it may be prudent to use similar methods, measures, and protocols in children and adolescents to allow cross-age comparison. Similarly, applying a multilevel approach in vicarious threat learning, using both self-reports and physiological measures, is especially important when studying youth and comparing age groups. Indeed, developmental differences in self-reports and physiological measures have already emerged; more specifically, younger children have more difficulty articulating what they learn observationally even though they physically respond differently to the threat and safety cues [9].

Future directions: As there is a renewed interest in vicarious threat learning among youth, there are many exciting new research avenues to explore. One important gap in the literature is the comparison of anxious and non-anxious youth in vicarious threat learning. Do certain downstream effects of vicarious threat learning, such as fear overgeneralization and behavioral avoidance of threat, contribute to the maintenance of acquired fear and anxiety symptoms? Moreover, as discussed, observer–demonstrator factors have yielded null or mixed results during vicarious threat learning. This is somewhat surprising, as it is reasonable to expect that differing social aspects (e.g., age, sex, empathy, relationship) would impact social threat learning in distinctive ways. More research should explore these specific observer–demonstrator questions of age congruency and modified empathy or trustworthiness during vicarious threat learning in youth. It may be particularly interesting to understand whether the information being passed (threat-related or otherwise) increases or diminishes the weight of observer-demonstrator factors. Does the potency of content overshadow the importance of certain observer–demonstrator factors during social threat learning? Another interesting question to answer is how neural functioning during vicarious threat learning manifests throughout development. The use of neuroimaging and other neurological measures (e.g., fNIRS, EEG/ERP) would greatly enhance our knowledge of specific developmental characteristics that may affect social threat learning. Finally, extrapolating vicarious threat learning and vicarious safety learning findings to the clinic would contribute to building new intervention programs to combat phobias and anxiety-related disorders in youth. A protocol based on positive modeling, such as ‘observational exposure therapy,’ may be used as a first step toward diminishing or even preventing suffering in a phobic or anxious developing population.
